# Decreasing Bio-Degradation Rate of the Hydrothermal-Synthesizing Coated Mg Alloy via Pre-Solid-Solution Treatment

**DOI:** 10.3390/ma10080858

**Published:** 2017-07-27

**Authors:** Dan Song, Cheng Li, Liwen Zhang, Xiaolong Ma, Guanghui Guo, Fan Zhang, Jinghua Jiang, Aibin Ma

**Affiliations:** 1 College of Mechanics and Materials, Hohai University, Nanjing 210098, China; songdancharls@hhu.edu.cn (D.S.); muzi6318@163.com (C.L.); Guo_guanghui@cypc.com (G.G.); zhangf416@gmail.com (F.Z.); 2 Suqian Research Institute of Hohai University, Suqian 223800, China; 3 Department of Materials Science and Engineering, North Carolina State University, Raleigh, NC 27695, USA; lzhang30@ncsu.edu (L.Z.); xma4@ncsu.edu (X.M.); 4 Department of Materials Science and Engineering, Kyushu University, Fukuoka 819-0395, Japan

**Keywords:** Mg alloy, conversion coating, solid solution treatment, microstructure, bio-degradation behavior

## Abstract

In this study, we report an effective approach, pre-solid solution (SS) treatment, to reduce the in-vitro bio-degradation rate of the hydrothermal-synthesizing coated Mg–2Zn–Mn–Ca–Ce alloy in Hanks’ solution. Pre-SS treatment alters the microstructure of alloys, which benefits the corrosion resistances of the substrate itself and the formed coating as well. The micro-galvanic corrosion between the secondary phase (cathode) and the α-Mg phase (anode) is relieved due to the reduction of the secondary phase. Meanwhile, coating formed on the SS-treated alloy was compacter than that on as-cast alloy, which provides better protection against initial corrosion.

## 1. Introduction

During the recent decades, Mg and its alloys have been widely studied due to its great potential in structural applications and human implant material [1,2,3,4,5,6,7,8,9,10]. Mg alloys exhibit similar densities and elastic modulus with human bone, as well as its excellent biocompatibility. However, their application in medical aspect is still limited due to the high bio-degradation rate in the body environment. Most of the Mg implants suffer severe degradation prior to the recovery of the injured tissues. Thus, it is of great practical significance to reduce the bio-degradation rate of Mg implants in order to prolong their service life. Various methods, such as alloying [11,12,13,14,15], heat treatment [16,17], plastic deformation [18,19,20,21,22], and surface treatment [23,24,25,26,27] have been employed to improve corrosion resistance of Mg implants. Great progress has been witnessed while further efforts are still imperative to further decrease biodegradation rate of the biomedical Mg alloys. 

Previously in our work, a novel kind of Mg–2Zn–Mn–Ca–Ce alloy was designed and fabricated [28]. It exhibited an improved corrosion resistance, while its bio-degradation rate was still not up to medical standards. An effective approach to reduce the bio-degradation rate of such an alloy was to hydrothermally synthesize a protective Mg(OH)_2_ coating [29]. However, the secondary phases of the Mg–2Zn–Mn–Ca–Ce alloy was found to deteriorate the coating compactness, leading to continuous micro-cracks on the coating layer [30]. Therefore, it is of great significance to reduce the secondary phase of the substrate, in order to i7mprove the coating integrity and its protection against corrosion. 

In this study, the cast Mg–2Zn–Mn–Ca–Ce alloy was a pre-solid-solution (SS) treated in order to reduce the secondary phase before the subsequent hydrothermal synthesis. The SS-treatment benefits both the protective efficiency of the coating and the corrosion resistance of the substrate, leading to further reduction in bio-degradation than before.

## 2. Experimental

### 2.1. Materials and Hydrothermal Synthesizing Processing

The Mg–2Zn–Mn–Ca–Ce alloy specimens with the size of 10 mm × 10 mm × 5 mm were cut from a cast ingot, whose chemical composition was listed in Table 1. The specimens were SS treated in an electric furnace with Argon protection. The specimens were heated at 500 °C for 24 h, followed by quench in the water. The hydrothermal synthesis processing was executed in stainless steel autoclave with a Teflon container (100 mL). The de-ionized water was poured into the Teflon container to 70% volume as the reaction solution. The reactor was heated via an electric furnace at 160 °C for 3 h. The hydrothermal-synthesizing time was counted after the furnace temperature reached the set temperature. Before synthesizing processing, all the specimens were polished with SiC papers up to 1800 grades, ultrasonically cleaned in acetone and ethanol for 5 min each, and dried in air. In each processing, one as-cast sample and one SS-treated sample were treated under the exactly same condition. The two processed samples were named as cast-coated sample and SS-coated sample, respectively.

### 2.2. Microstructure Characterization

The surface and cross-sectional micro-morphologies of the coatings, as well as the microstructure of the substrate Mg alloys, were examined by scanning electron microscope (SEM, Sigma 500, Zeiss, Heidenheim, Germany and Verios 460L, FEI, Hillsboro, OR, USA). Prior to the observation, all the samples were coated by gold. The element distribution of the cast and SS-treated alloy were characterized by the energy dispersive X-ray spectrometer (EDS, OXFORD instrument, Oxford, Oxfordshire, UK). X-ray diffraction (XRD) analysis of the coated sample was performed using a Bruker D8 Advance diffractometer (Bruker AXS, Karlsruhe, Germany) with Cu Kα1 radiation. The θ–2θ diffraction patterns were scanned from 10° to 90° with a scanning rate of 2°·min^−1^.

### 2.3. Corrosion Tests

In-vitro bio-degradation behaviors of the coated samples and the substrate samples were studied by hydrogen-evolution immersion test and electrochemical tests at 37 °C. The substrate samples were prepared by mechanical polishing of the coated samples to remove the coating layer. Later, two substrate samples were named as cast-substrate and SS-substrate, respectively. Hanks’ solution was selected as the simulated body fluid, whose chemical composition was listed in the Table 2. The Hanks’ solution was renewed every single day to keep the corrosion environment consistent.

Before the hydrogen evolution immersion test, the coated samples and the substrate samples were molded in epoxy with a squared exposure of 1 cm^2^. All exposed surfaces were cleaned by acetone and ethanol prior to tests. The evolved hydrogen was collected and recorded with different immersion time, which was converted to hydrogen evolution rate. After that, the corrosion morphologies of the samples were observed via a digital microscope (Hirox, KH-7700, Hackensack, NJ, USA) and the SEM (Sigma 500, Zeiss, Heidenheim, Germany and Auriga Crossbeam Microscope, Zeiss, Heidenheim, Germany).

Electrochemical tests were conducted via a Parstat 2273 (Princeton, Oak Ridge, TN, USA) advanced potentiostat with a three-electrode cell. The samples were prepared using the same method in immersion tests and then were connected by the copper wire for electrochemical tests. The potentiodynamic polarization (PDP) test and electrochemical impendence spectroscopy (EIS) test were systematically conducted. Before the PDP and EIS test, the samples were pre-immersed in the solution for 1 h to reach the stable open circuit potential. The PDP tests were performed at a scan rate of 1 mV·s^−1^. The frequency range of EIS tests were from 10 kHz to 10 mHz, and the applied amplitude of sinusoidal potential was 20 mV.

## 3. Results and Discussion

### 3.1. Microstructure Observation of the Substrate Alloys

**Figure 1:**
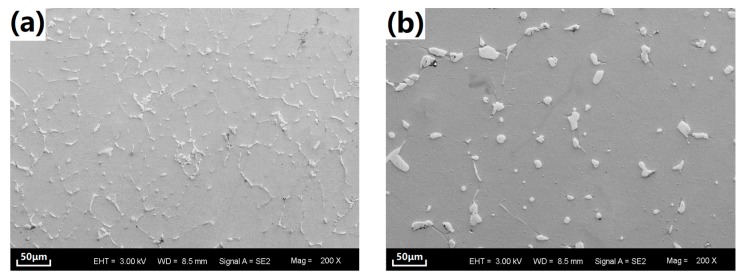
SEM microstructure of the Mg alloy substrates. (**a**) as-cast alloy; (**b**) solid solution (SS) treated alloy.

Figure 1 shows typical SEM microstructure of the substrates in as-cast alloy and SS-treated alloy, respectively. The as-cast alloy shows two major microconstituents: α-Mg phase with relatively small grain size and secondary phases. According to our previous work, the secondary phases have been identified as Ca_2_Mg_6_Zn_3_, Mg_2_Ca, and Mg_12_CeZn phase, which are net-like and distributed along the grain boundaries of the α-Mg phase [28]. In contrast, the α-Mg phase grains are larger in the SS-treated sample. Meanwhile, the former secondary-phase net structure was changed to isolated secondary-phase particles. Those particles were coarse and distributed both inside of the α-Mg grains and along the grain boundaries. During the heating, secondary-phase particles were triggered to diffuse to the α-Mg grains leading to their disappearance and their decrease in volume fraction. Determined by image analysis via MATLAB (MathWorks, Natick, MA, USA), the secondary-phase volume fractions of the as-cast and ss-treated alloy are 3.4% and 2.5%, respectively. Meanwhile, the remaining secondary-phases tend to agglomerate as coarse particles in order to reduce the interface energy. 

**Figure 2:**
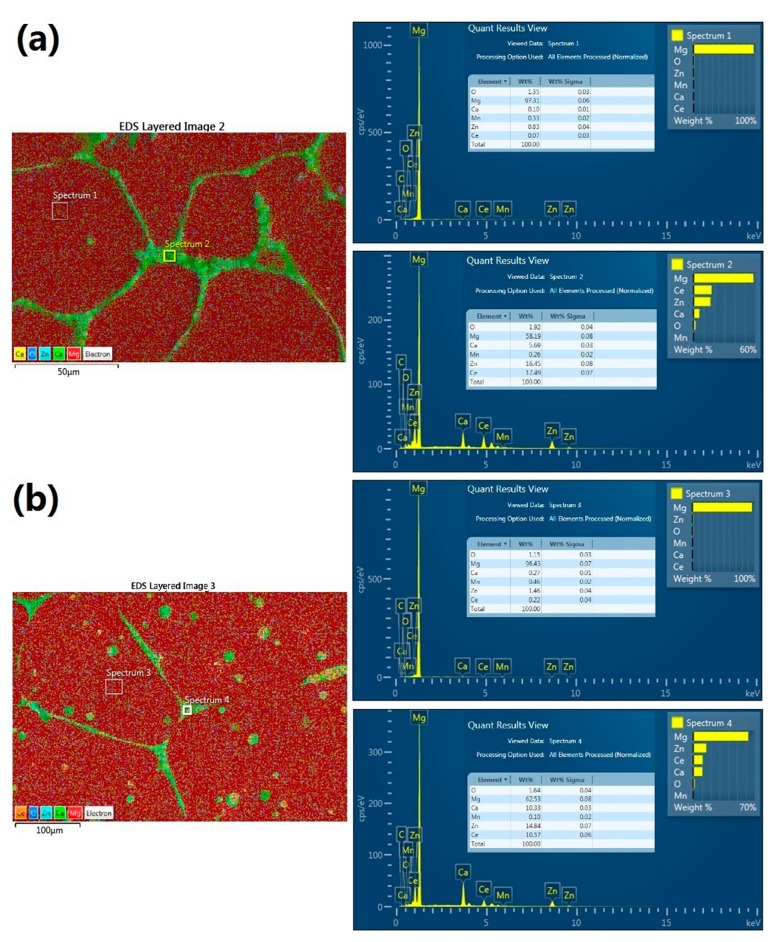
Energy dispersive X-ray spectrometer (EDS) analysis of the cast and SS substrate alloy. (**a**) as-cast alloy; (**b**) SS treated alloy.

EDS analysis was conducted to study the elements distribution change in the alloy after SS treatment. As shown in Figure 2a, the chemical content of the α-Mg phase is about 97.31 Mg, 0.83 Zn, 0.33 Mn, 0.1 Ca, and 0.07 Ce (wt %) and the chemical content of the primary secondary phase is about 58.19 Mg, 16.45 Zn, 0.26 Mn, 5.69 Ca, and 17.49 Ce (wt %) in as-cast alloy. While in Figure 2b, the chemical content of the α-Mg phase is about 96.43 Mg, 1.46 Zn, 0.46 Mn, 0.27 Ca and 0.22 Ce (wt %) and the chemical content of the primary secondary phase is about 62.53 Mg, 14.84 Zn, 0.1 Mn, 10.33 Ca, and 10.57 Ce (wt %) in SS-treated alloy. Clearly, the alloying-element atoms diffused from the secondary phases into the α-Mg phase during the treatment. As a result, the alloying-element contents were increased in α-Mg phase but decreased in the residential secondary-phase particles while the Mg content changed oppositely. Moreover, the composition gap between the α-Mg phase and secondary phases is slightly reduced in the SS-treated alloy.

### 3.2. Microstructure Observation of the Coatings

**Figure 3:**
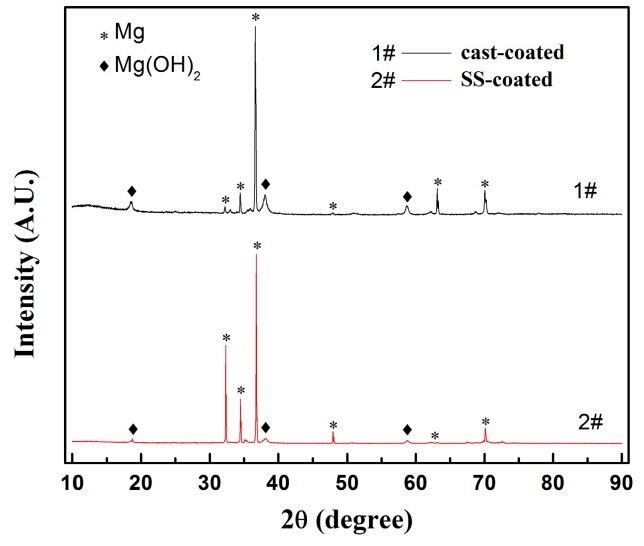
X-ray diffraction patterns of the coated samples.

X-ray diffraction was employed to analyze the phases of the coated samples. As shown in Figure 3, both coated samples showing the typical Mg patterns and Mg(OH)_2_ patterns. As found in the previous work [28], these two patterns correspond to the Mg alloy substrate and the coating layer, respectively. Because of the trivial volume fraction and the sensitivity of the X-ray instrument under the current settings, no XRD pattern of the secondary phase was detected in either of the two samples. In addition, the Mg(OH)_2_ peak intensity is slightly lower in the SS-coated sample than in the cast-coated sample. Given the same X-ray scanning depth, the relatively weak Mg(OH)_2_ peak is an evidence to the thinner coating layer of the SS-coated sample.

**Figure 4:**
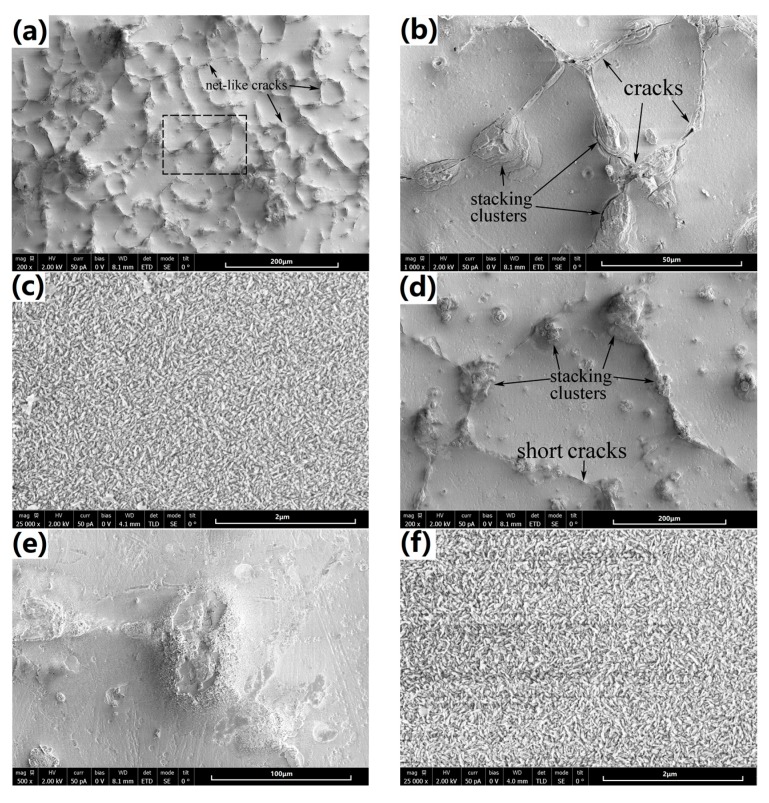
Surface morphologies of the 3-hours coated samples observed at different magnifications. (**a**–**c**) are the cast-coated sample; (**d**–**f**) are the SS-coated sample.

Figure 4 shows the SEM surface morphologies of the coatings synthesized on the cast and SS-treated substrate alloy. As seen in Figure 4a (low-magnification) and Figure 4b (local high-magnification from the mark region in 4a), the coating on the cast alloy (named cast-coating briefly) presented net-like cracks. Particularly, these cracks appear more likely along the α-Mg phase grain boundaries or the secondary-phase net structure. Additionally, the specific stacking clusters are observed to primarily distribute on the secondary phases. Those stacking clusters are loose so that cracks prefer to originate from them. On the other hand, the coating layer on the α-Mg matrix is more compact, and free of micro cracks, as shown in highly magnified image in Figure 4c. The coating layer is composed of nano-scale structures, which are reported as hexagonal flake Mg(OH)_2_ crystal units before [25,27]. In contrast, only a few short cracks can be found in the coating on the SS-treated alloy (named SS-coating briefly), which is shown in Figure 4d with the same magnification to Figure 4a. Interestingly, the stacking clusters distribute not only on the α-Mg grain boundaries, but also inside the grain in SS-treated alloy. Given the SEM observation in Figure 1b, it is speculated that the stacking clusters inside the α-Mg grains are generated on the remaining secondary-phase particles. Note that the stacking clusters are generally larger than their counterparts in as-cast alloy. This is reasonable since secondary-phase particles are relatively large in the SS-treated alloy. As shown in Figure 4e, no obvious micro-cracks can be found on the stacking clusters, which show better compactness than that of cast-coated sample. Figure 4f shows that the coating layer on α-Mg matrix exhibits very similar microstructure to that in the cast-coated sample. Based on the above description, one can confirm that the cracks are significantly decreased on the SS-coating, which endows its better compactness. 

**Figure 5:**
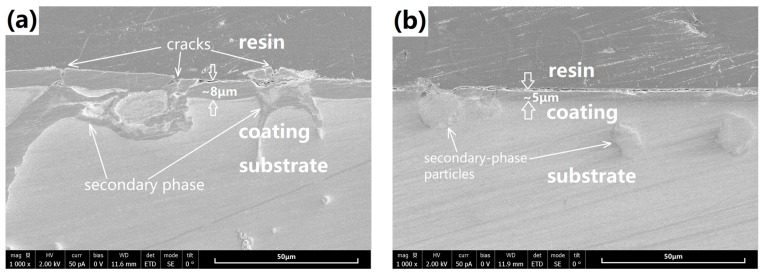
Cross-sectional morphologies of the 3-hours coated samples: (**a**) cast-coated sample; (**b**) SS-coated sample.

Figure 5 shows the cross-sectional morphologies of the coated samples. Both coatings were well bonded to the substrate, free of micro-cracks at the substrate/coating interface. Clearly, SS-coating is about 5 μm thick, obviously thinner than the cast-coating (about 8 μm). Note that the coating on the α-Mg matrix takes most part of the coating, the much thinner SS-coating should have a relationship with the microstructure and chemical composition of its α-Mg phase. Beside the coating thickness, differences can also be found in the coating compactness. Cracks can be easily found in the cast-coating, particularly above the secondary phases of the substrate alloy, which were marked by the white arrows in the Figure 5a. In contrast, besides the thinner coating covered on the secondary-phase particles, the SS-coating is much compacter in the cross-sectional view. 

### 3.3. In-Vitro Degradation Behavior 

The synthesized Mg(OH)_2_ coating layer is believed to provide a barrier against the penetration of an aggressive medium. Thick and compact coating will provide better protection against corrosion. Once the aggressive medium reaches the coating/matrix interface, corrosion damage will occur in the substrate alloy as well. The overall corrosion reaction of Mg in many aggressive mediums (including the real human body environment and the simulated body fluid) can be expressed as follows [31,32].

Mg + H_2_O → Mg^2+^ + 2OH^−^ + H_2_ (1)

The corrosion of the substrate Mg alloy leads to not only the materials degradation, but also the hydrogen evolution. The generated hydrogen bubbles will, in turn, destroy the integrity of the coating and weaken its protection effect, accelerating the corrosion damage in the substrate Mg alloy. In other words, once corrosion locally penetrates the coating to the substrate, the degradation processing of the coated Mg alloy will become self-accelerating. 

**Figure 6:**
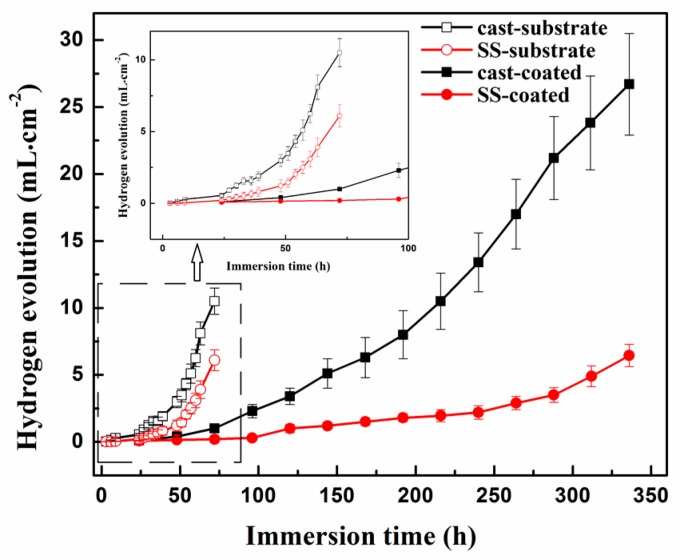
Hydrogen-evolution rates of the substrate Mg alloys and the coated samples in Hanks’ solution.

The hydrogen-evolution rates of the substrate Mg alloys and the coated samples in Hanks’ solution were shown in Figure 6. Firstly, the hydrogen evolution of the substrates is about 5-times faster than that of the coated samples after 3-days corrosion, which indicate the significant protective effect of the coating. Given their great difference in degradation rate, the substrates were tested for a shorter period (72 h) while the coated samples were tested for a longer period (336 h). Regarding the two substrates, the SS-treated substrate (named SS-substrate briefly) exhibits lower hydrogen-evolution rate than that of the cast substrate (named cast-substrate), indicating the better corrosion resistance of the SS-substrate. Note that this phenomenon is more remarkable during the initial immersion period. On the other hand, the SS-coated samples show a significantly lower hydrogen evolution rate during the whole immersion period. The total generated hydrogen of the SS-coated sample is nearly one fifth of the cast-coated sample. Another difference is the incubation period, prior to which no detectable hydrogen evolution had been generated. The longer incubation period can be regarded as the retarded corrosion initiation of the sample. The incubation period of the SS-coated sample is about 96 h, almost double that of the cast-coated sample (48 h). Apart from the difference, both coated samples presented increased hydrogen-evolution rate as the immersion period increased. This phenomenon should be caused by the accelerated degradation in substrates and the gradually destroyed coating integrity.

**Figure 7:**
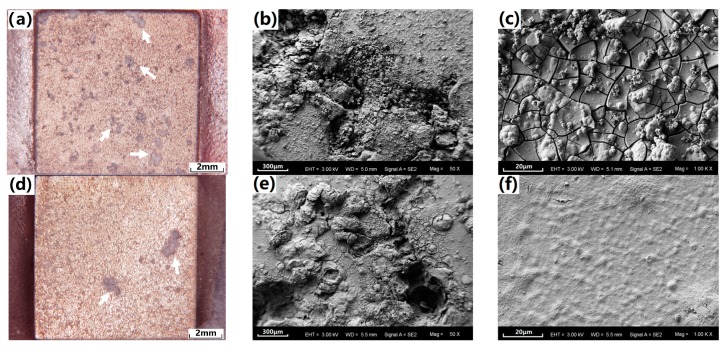
Optical and SEM corrosion morphologies of the coated samples after hydrogen-evolution immersion in Hanks’ solution for five days. (**a**–**c**) are cast-coated sample; (**d**–**f**) are the SS-coated sample.

Figure 7 presents the optical and SEM corrosion morphologies of the coated samples after hydrogen-evolution immersion in Hanks’ solution for five days. As seen in Figure 7a,b, both samples present typical localized corrosion morphologies under optical micrographs. Given the number and size of the corrosion spots (as marked by the white arrows), the corrosion damage of the SS-coated sample was clearly less than that of the cast-coated sample. As seen in Figure 7b,e, the SEM morphologies of the corrosion spots in the two samples showed similar corrosion damage in both the coating layer and the substrate alloys. The corrosion spots were covered by the loose corrosion production, and the corrosion damage has propagated in the depth direction of the substrate. However, a great difference can be found in the non-serious corroded zone of the coating via SEM, which is shown in Figure 7c,f. The cast-coating has been seriously cracked while the SS-coating still kept integrated, no cracks can be found. The mass of cracks on the cast-coated sample provided channels for the direct penetration of the corrosive medium. In contrast, the SS-coating can still provide sufficient prevention due to the better integrity and compactness of the coating.

**Figure 8:**
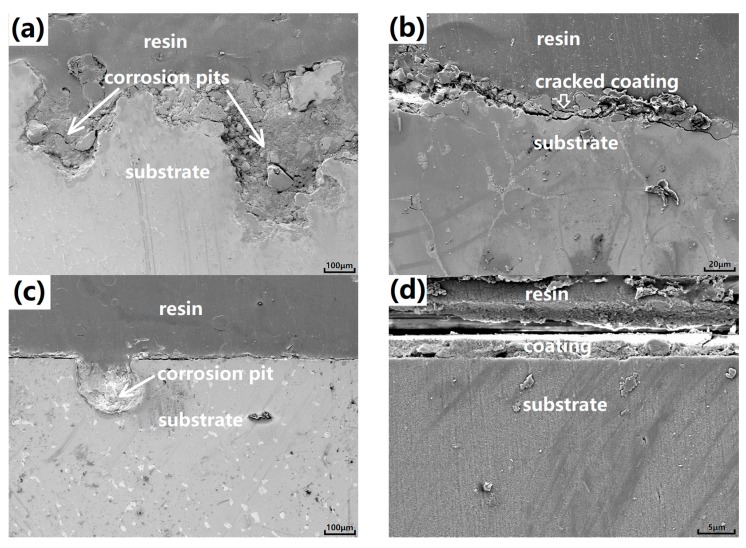
SEM cross-sectional corrosion morphologies of the coated samples after hydrogen-evolution immersion in Hanks’ solution for five days. (**a**,**b**) are the cast-coated sample; (**c**,**d**) are the SS-coated sample.

Figure 8 presents the SEM cross-sectional corrosion morphologies of the coated samples after hydrogen-evolution immersion in Hanks’ solution for five days. Figure 8a,c is typical corrosion pits related to the localized corrosion (corrosion spots, as marked by the white arrows in Figure 7a,d.) of the cast-coated and ss-coated samples. Clearly, the corrosion pits of the SS-coated sample are of less size and depth compared to that of the cast-coated sample, which indicated its milder localized corrosion damage. Figure 8b,d is cross-sectional morphologies of the coatings away from the localized corrosion pits. Due to the protection of the coating, there was no obvious localized corrosion found in the substrate under the coating of both samples. The coating morphologies of both samples have been greatly changed from the cross-sectional view, especially the cast-coated one. After corrosion, the cast-coating has been completely cracked while the ss-coating is still relatively intact, although the ss-coating seems to be less compact compared to its as-synthesized state (as seen in the Figure 5b). Judged from both the top and the cross-sectional views, one can envision that the ss-coating will provide better protection during the further immersion corrosion due to its better compactness. 

**Figure 9:**
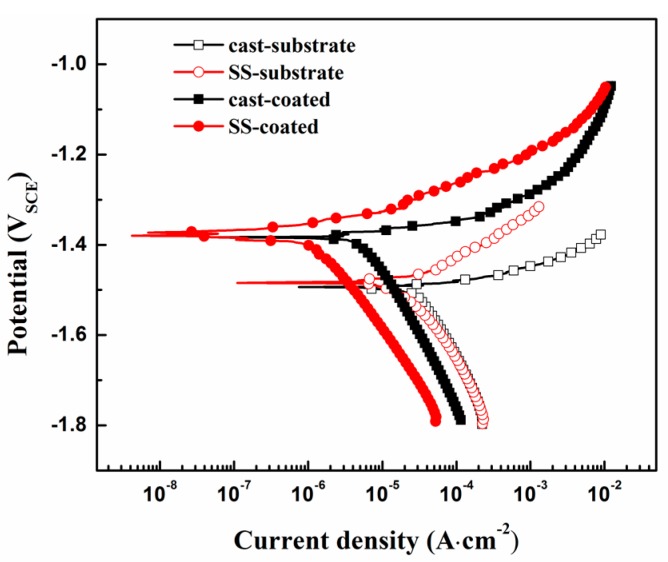
Polarization curves of the coated and substrate samples immersed in Hanks’ solution.

Figure 9 presents the PDP curves of the coated and substrate samples in the Hanks’ solution. The corrosion potentials (*E*_corr_) the corrosion current densities (*I*_corr_) were derived directly from the PDP curves by the Tafel extrapolation method, and were summarized in Table 3. The fitted cathodic slope (*β*_c_) and anodic slope (*β*_a_) and the calculated polarization resistance (*R*_p_) were also listed in the Table 3. The *R*_p_ values were calculated according to the Equation (2) [33] as follows:

(2) $$R_{P}=\frac{\beta_{a}\beta_{c}}{2.3\left(\beta_{a}+\beta_{c}\right)I_{corr}}.$$

Generally, the coated sample had nobler *E*_corr_ values and smaller *I*_corr_ values than the substrates, indicating the better corrosion resistance of coatings as reported earlier [34]. For two different kinds of substrates, they have similar *E*_corr_ but different *I*_corr_ values. The *I*_corr_ of the SS-substrate is about 1.64 × 10^−5^ A·cm^−2^, smaller than that of the cast-substrate (about 2.71 × 10^−5^ A·cm^−2^), indicating its better corrosion resistance. In terms of the two coated samples, the *I*_corr_ of the SS-coated one is about 1.27 × 10^−6^ A·cm^−2^, about a quarter of the cast-coated one (about 4.56 × 10^−6^ A·cm^−2^). Smaller *I*_corr_ value indicated much lower degradation rate and greatly relieved corrosion damage in the substrate.

**Figure 10:**
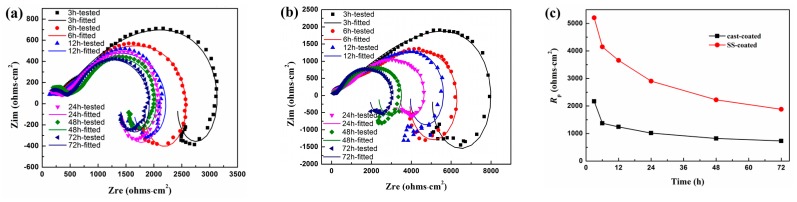
Electrochemical impendence spectroscopy (EIS) characteristic parameters of the coated samples. (**a**,**b**) are the EIS Nyquist plots of the cast-coated and SS-coated samples respectively; (**c**) is the fitted *R*_p_ value curves respectively.

EIS was further conducted to study the stability of the coating in the Hanks’ solution. Figure 10 shows EIS *Nyquist* plots of the coated sample after in-vitro immersion for different periods. All *Nyquist* plots were composed of two capacitive arcs and one inductive arcs. Two capacitive arcs are the typical *Nyqusit* plot of many coated samples because the high/low-frequency arcs are related to EIS signal responded from the substrate/coating, respectively [35]. Generally speaking, a larger capacitive arc represents better corrosion resistance. It is noteworthy that the SS-coated samples present larger low-frequency capacitive arcs during the whole testing period.

The *R*_s_(*C*_f_(*R*_p_(*C*_dl_*R*_t_(*R*_L_L)))) equivalent circuit was used to fit the EIS plots, where *R*_s_ is the electrolyte solution resistance, *R*_p_ and *C*_f_ represent the microporous resistance and capacitance of the Mg(OH)_2_ coating, *C*_dl_ and *R*_t_ represent the double layer capacitance and the charge transfer resistance of the substrate, *R*_L_ and *L* represent the inductive loop [23]. Figure 10c shows the fitted *R*_p_ of the both kinds of coated samples. Obviously, the *R*_p_ values decreased with the immersion time, indicating the corrosion damage in both coatings and substrates. However, the SS-coated samples always keep the larger *R*_p_ values than that of the cast-coated samples during the whole immersion period. The larger *R*_p_ values of the SS-coated sample indicate the better integrity and protection of its coating.

### 3.4. Positive Effect of Pre-SS Treatment on the Coating and the Substrate

Based on the discussions above, it is found, collectively, that the SS-coated sample has greatly reduced the degradation rate in comparison to the cast-coated sample. The better performance of the SS-coated sample should stem from its improved corrosion resistance of the substrate and the better protection of the coating. 

It is not difficult to understand the better corrosion resistance of the SS-substrate, many references have reported the positive effect of SS treatment on improving the corrosion resistance of the Mg alloys [36,37,38]. During the corrosion processing, the secondary phase is the cathodic phase and can be kept stable, while the α-Mg phase is the anodic phase and will be preferentially corroded. As shown in the SEM microstructure, the volume fraction of the secondary phase was greatly reduced after SS treatment, leading to the significant decrease in the micro-galvanic corrosion between these two phases. This is the primary reason for the improved corrosion resistance of the SS-substrate. 

In view of the lower degradation rates of the coated samples, both coatings provide sufficient protection. However, the SS-coating exhibits even higher protective efficiency than the cast-coating in our observations. Generally, the protection power of a coating against corrosion for this Mg alloy is largely determined by its thickness and compactness. The actual performance is therefore the comprehensive results given both characters. In this study, it is found the SS-coating is thinner but compacter and turns out to provide better protection efficiency than the cast-coating. In other words, compactness is probably prioritized over thickness regarding protection performance of the coating on the alloy. This is reasonable because the defects like micro cracks provide the shortcut for the penetration of the corrosive medium. The coating fails to fulfill its protective functionality after such penetration no matter how thick it is. The formation of such defective coating is closed related to the secondary phase distribution and morphology [29,30]. After pre-SS treatment of the substrate, the disappearing of secondary-phase net-like structure and decrease in volume fraction was achieved, and the net-like cracks of the coating were suppressed accordingly.

**Figure 11:**
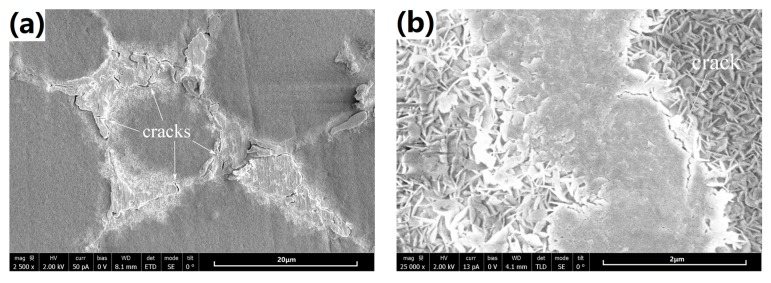

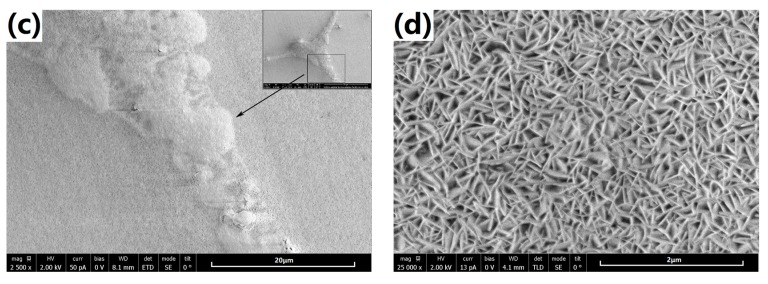
Surface morphologies of the 1-hour coated samples observed at different magnification. (**a**,**b**) are the cast-coated sample; (**c**,**d**) are the SS-coated sample.

For better understanding the effect of SS-treatment on the coating formation, a 1-h synthesized coating was carefully examined by SEM, as shown in Figure 11. Clearly, as for the cast-coated sample, the coatings on the α-Mg phase and the secondary phase present extremely different stacking structures of Mg(OH)_2_ crystal units. In addition, micro cracks are frequently observed along the boundary of the α-Mg phase and secondary phase, which may be caused by the different volume shrinkage ratio of both phases of the coating during the drying processing after hydrothermal synthesizing. However, just as the SS-coated sample, the coatings present the similar stacking structure of Mg(OH)_2_ crystal units on the α-Mg phase and the secondary phase. Importantly, no micro cracks can be found. This phenomenon may have a close relationship to the homogenization process during SS treatment. Bear in mind that the composition difference of two phases was reduced of the SS-treated substrate, as shown in Figure 2. This may lead to the similar shrinkage behavior during drying. Also, relatively lower Mg and higher alloying concentrations probably give rise to a thinner coating eventually on the SS-treated substrate.

## 4. Conclusions

The bio-degradation rate of the hydrothermal-synthesizing coated Mg–2Zn–Mn–Ca–Ce alloy in Hanks’ solution was greatly reduced via pre-solid-solution treatment of the substrate. The better performance of the SS-coated sample was benefited from both the better protection of the coating and the improved corrosion resistance of the substrate. 

Coating formed on the SS-treated alloy was thinner, but compacter than that on the as-cast alloy, and the coating compactness probably plays a more important positive role in the coating’s protective efficiency. Elimination of the secondary-phase net-like structure of the substrate suppressed the continuous cracks of the coating, and endows the coating enhanced compactness. The thinner coating was primarily attributed to the lower Mg content and higher alloying-elements content of the α-Mg phase of the SS-substrate. 

SS-substrate presented better corrosion resistance compared to the cast-substrate. The micro-galvanic corrosion between the secondary phases (cathode) and the α-Mg phase (anode) was relieved due to the greatly reduced secondary phases.

## Figures and Tables

**Table 1:** Chemical composition of Mg–2Zn–Mn–Ca–Ce alloy (wt %).

Zn	Mn	Ca	Ce	Mg
2.00	0.50	1.02	1.35	balance

**Table 2:** Chemical composition of Hanks’ solution.

Solution	Chemical Composition (mmol·L^−1^)							
	NaCl	CaCl_2_	MgSO_4_	KCl	KH_2_PO_4_	Na_2_HPO_4_	D-Glucose	NaHCO_3_
Hanks’	137	1.261	0.814	5.33	0.44	0.338	5.56	4.17

**Table 3:** Electrochemical parameters of the samples obtained via potentiodynamic polarization (PDP) tests.

Samples	*E*_corr_ (V)	*I*_corr_ ( A·cm^−2^)	*β*_a_ (V·dec^−1^)	*β*_c_ (V·dec^−1^)	*R*_p_ (ohm·cm^2^)
Cast-coated	−1.38	4.56 × 10^−6^	0.041	0.242	3343
SS-coated	−1.37	1.27 × 10^−6^	0.060	0.233	16,334
Cast-substrate	−1.49	2.71 × 10^−5^	0.038	0.256	531
SS-substrate	−1.48	1.64 × 10^−5^	0.058	0.227	1225
